# The Impact of Obesity on C1q/TNF-Related Protein-9 Expression and Endothelial Function following Acute High-Intensity Interval Exercise vs. Continuous Moderate-Intensity Exercise

**DOI:** 10.3390/biology11111667

**Published:** 2022-11-15

**Authors:** Brandon G. Fico, Ryan S. Garten, Michael C. Zourdos, Michael Whitehurst, Peter J. Ferrandi, Katelyn M. Dodge, Gabriel S. Pena, Alexandra A. Rodriguez, Chun-Jung Huang

**Affiliations:** 1 Department of Kinesiology, University of Wisconsin-Madison, Madison, WI 53706, USA; 2 Department of Kinesiology and Health Sciences, Virginia Commonwealth University, Richmond, VA 23284, USA; 3 Exercise Biochemistry Laboratory, Department of Exercise Science and Health Promotion, Florida Atlantic University, Boca Raton, FL 33431, USA; 4 College of Graduate Health Sciences, The University of Tennessee Health Science Center, Memphis, TN 38163, USA; 5 Department of Kinesiology, University of Maryland, College Park, MD 20742, USA

**Keywords:** high-Intensity interval exercise, endothelial function, flow-mediated dilation, C1q-TNF-related protein-9, obesity

## Abstract

**Simple Summary:**

Lifestyle modifications such as diet and exercise are a first-line defense to promote health in individuals with obesity. High-intensity interval exercise has recently gained popularity as a time-effective exercise modality. As such, this work compared the acute exercise induced benefits on vascular health between high-intensity interval exercise and traditional continuous moderate-intensity exercise. We found that both exercise modalities lead to improvements in indicators of vascular health, with some enhancements lasting up to 2 h following exercise. Therefore, high-intensity interval exercise is a time-effective strategy to improve vascular health similarly to traditional continuous moderate-intensity exercise in individuals with obesity.

**Abstract:**

C1q-TNF-related protein-9 (CTRP9) increases endothelial nitric oxide synthase and reduces vasoconstrictors. There is limited information regarding exercise-mediated CTRP9 in obesity. The purpose of this study was to compare high-intensity interval exercise (HIIE) and continuous moderate-intensity exercise (CME) on the CTRP9 response and an indicator of endothelial function (FMD) in obese participants. Sixteen young male participants (9 obese and 7 normal-weight) participated in a counterbalanced and caloric equated experiment: HIIE (30 min, 4 intervals of 4 min at 80–90% of VO_2_ max with 3 min rest between intervals) and CME (38 min at 50–60% VO_2_ max). Serum CTRP9 and FMD were measured prior to, immediately following exercise, and 1 h and 2 h into recovery. CTRP9 was significantly increased immediately following acute HIIE and CME in both groups (*p* = 0.003). There was a greater CME-induced FMD response at 2 h into recovery in obese participants (*p* = 0.009). A positive correlation between CTRP9 and FMD percent change was observed in response to acute CME when combined with both obese and normal-weight participants (r = 0.589, *p* = 0.016). The novel results from this study provide a foundation for additional examination of the mechanisms of exercise-mediated CTRP9 on endothelial function in individuals with obesity.

## 1. Introduction

Obesity is a national epidemic in the United States as its incidence has steadily increased for the last three decades and continues to rise [1]. The metabolic profile associated with obesity is linked to an augmented risk of inflammatory diseases, such as diabetes and cardiovascular disease [2]. The development of these cardiovascular consequences include infiltration of inflammatory leukocytes (e.g., macrophages) to the vessel wall and apoptosis of smooth muscle cells, leading to endothelial dysfunction [3,4]. The subsequent impairment of endothelium-dependent vasodilation or vasomotor function is one of the first subclinical stages in the atherosclerotic process [5].

A method of assessing endothelial function and vasomotor reactivity is flow-mediated dilation (FMD), which stimulates the release of endothelial relaxing factors (e.g., nitric oxide [NO]), resulting in vasodilation [6]. Additionally, obesity-related inflammation and oxidative stress have a destructive effect on the endothelium via down-regulation of vasodilatory signaling pathways [7,8], such as the AMP-activated protein kinase (AMPK) mechanism [9]. Specifically, AMPK promotes endothelial cell NO synthase (eNOS) phosphorylation through direct or indirect protein kinase B (Akt) activation [10]. A recent novel adipocytokine, C1q-TNF-related protein-9 (CTRP9), increases eNOS activation via the AMPK-Akt-eNOS mechanism in human umbilical vein endothelial cells [9]. Moreover, CTRP9 is a paralog of adiponectin that activates AMPK, Akt, and p44/42 MAPK signaling pathways [11]. Importantly, CTRP9 is down-regulated in obese mice [10] and patients with insulin resistance [12,13]. Serum CTRP9 is also inversely correlated with visceral fat in humans [12]. Interestingly, the level of circulating CTRP9 is elevated in obese individuals but significantly decreases following weight loss surgery, suggesting a compensatory role of CTRP9 in obesity [14]. Taken together, these findings may support the important role of CTRP9 as a potential mechanism to attenuate obesity-induced endothelial dysfunction.

Moderate intensity exercise has been shown to reduce inflammation [15], while high-intensity interval exercise (HIIE) improves endothelial function in cardiac patients more than continuous moderate-intensity exercise (CME) [16,17]. A greater improvement in aerobic capacity, as evidenced by increased peak oxygen consumption was observed in patients with coronary artery disease following ten weeks of HIIE training when compared to CME [16]. In comparison of CME training, HIIE training reduced resting blood pressure and arterial stiffness as an indication of improved vascular function in hypertensive patients [18]. A meta-analysis concluded that HIIE training elicits a greater improvement of FMD compared to CME training [19]. Regarding the time course of FMD response, an elevation was observed up to two hours into recovery following acute HIIE, whereas CME remained unchanged in healthy adolescents [20]. Moreover, acute high-intensity continuous exercise showed a significant increase in FMD in normal-weight but not obese participants, with no difference observed in response to acute moderate-intensity continuous exercise [21]. Acute HIIE has been demonstrated to increase CTRP9 immediately following exercise in healthy young participants [22]. However, the exact mechanisms regarding obesity-mediated FMD and CTRP9 responses with either HIIE or CME remains to be elucidated. Therefore, the primary purpose of this study was to utilize acute HIIE as a time-effective exercise modality to understand CTRP9 response and its possible relationship with an indicator of endothelial function (FMD) when compared to CME in obese vs. normal-weight participants. We hypothesized that HIIE would be as effective as CME at increasing CTRP9 and FMD following exercise.

## 2. Methods

### 2.1. Participants

Sixteen (9 obese and 7 normal-weight) relatively healthy young male participants participated in this study. Participants with a body mass index (BMI) above 30 kg/m^2^ were classified as obese, and those with a BMI between 18.5 and 24.9 kg/m^2^ were classified as normal-weight. All participants completed an informed consent form, a medical history questionnaire, and 7-day physical activity record prior to data collection. The study was approved by the Florida Atlantic University’s Institutional Review Board and were performed according to the Declaration of Helsinki, with written informed consent from each participant.

Participants were excluded from the study if they had any known or suspected cardiovascular, metabolic, rheumatologic, or other inflammatory disease. Participants were also excluded from the study if they were taking any medication or supplements, users of tobacco products (cigarettes, cigars, chewing tobacco, vapors), or if they consumed an average of more than ten alcoholic beverages per week. These exclusion criteria were determined using the health history questionnaire. Participants fasted overnight for at least eight hours and abstained from alcohol, caffeine intake, and intense physical activity for at least 24 h prior to each lab visit.

### 2.2. Experimental Protocol

All participants performed three exercise protocols, with a minimum of one week separating each session. Participants started each study visit at the Exercise Biochemistry Laboratory between 7:00–7:30 a.m. During the first visit, following completion of the informed consent form, medical history questionnaire, and 7-day physical activity questionnaire, height and weight was measured (SECA 769, Chino, CA, USA), we also measured each participant’s hip and waist circumference to determine waist-to-hip ratio. Additionally, after 20 min of sitting, resting heart rate was recorded using a heart rate monitor (Polar T31, Polar Electro, Kempele, Finland) and blood pressure was assessed using a sphygmomanometer (752M-Mobile Series, American Diagnostic Corporation, Hauppauge, NY, USA). Next, blood sampling was performed by a trained phlebotomist (B.G.F) using standard aseptic techniques. A closed IV catheter system (BD Nexiva 20 GA, REF 383516, Franklin Lakes, NJ, USA) was inserted in the superficial vein of the upper arm in the antecubital space. Approximately 30 mL of blood were collected into specific collection tubes for subsequent analysis. Prior to the beginning of the exercise protocol, participants rested supine for 20 min before the assessment of their resting (baseline) FMD measurement using an ultrasound (Phillips iU22, Foster City, CA, USA).

Participants completed a graded exercise test using a treadmill (Norditrack X11i) designed to assess maximal oxygen consumption (VO_2_ max) measured by open-circuit spirometry (ParvoMedics Metabolic Measurement System (ParvoMedics, Sandy, UT, USA)) and maximal heart rate (HR_max_). The maximal exercise protocol started with a three-minute warm up at 60% age-predicated maximal heart rate (HRmax), followed by an increase in speed until 80% HR_max_. Subsequently, the grade was increased by 2% every two minutes until attainment of VO_2_ max. The validation of VO_2_ max was verified by either the primary criterion of a plateau in VO_2_ or 2 of the 3 secondary criteria are achieved. The secondary criteria used were (1) reaching predicted maximal heart rate, (2) achieving a respiratory exchange ratio of >1.15, and (3) reporting a rating of perceived exertion (with the 15-point Borg Scale) of 19 or 20. Importantly, this perceived exertion scale was used to measure the perception of stress each minute during exercise. If participants reported a difficulty to maintain exercise intensity, then the exercise testing was terminated.

During the second and third visits, participants were randomly allocated to participate in either HIIE or CME following a previously validated treadmill protocol [16,23]. The HIIE consisted of 30 min of exercise, including a 5 min warm-up period of 50–60% VO_2_ max (65–75% HR_max_), 4 intervals of 4 min at an intensity that elicits 80–90% VO_2_ max (85–95% HR_max_). Between the intervals the participants walked for 3 min at 50–60% VO_2_ max (65–75% HR_max_), with no active recovery following the last high-intensity bout. To achieve an isocaloric protocol between the exercise protocols, CME consisted of 38 min at 50–60% VO_2_ max (65–75% of HR_max_), which equated total oxygen consumption (VO_2_) across time of exercise as previously described [16]. Blood collection and FMD measurements for both visits 2 and 3, followed the same protocol as the first visit.

### 2.3. Blood Sampling

During each blood draw, 30 mL of the blood were collected with 5 mL into a serum separation tube (SST) for serum protein analysis and centrifuged for 10 min at 1300× *g* at room temperature. The serum was collected and stored in aliquots at −80 °C for subsequent analysis by enzyme-linked immunosorbent assays (ELISA). Serum CTRP9 was analyzed using (Cloud-Clone Corp., Houston, TX, USA).

### 2.4. FMD Measurement

The FMD measurement and protocol followed previously established guidelines [24]. The brachial artery was identified and imaged longitudinally using a Phillips L9-3 broadband 9.0 MHz vascular probe on the medial upper arm 2–10 cm above the antecubital fossa near the level of the heart. Landmarks were identified to ensure the same location for all repeated FMD measurements. Additionally, with-in each visit the probe was outlined to ensure identical placement for each time-point. Diameter and blood flow velocity were recorded in Digital Imaging and Communications in Medicine (DICOM) format using a duplex mode of ultrasound that allows simultaneous B-mode imaging for diameter measurements and Doppler for blood velocity (shear rate) using a Philips iU22 ultrasound. The insonation angle was set to 60° and gate width was adjusted for an accurate measurement of blood velocity as described by Harris et al., 2010.

Participants laid supine for 20 min prior to baseline FMD measurements, following acclimatization baseline measurements were recorded for 1 min. A blood pressure cuff (WelchAllyn 406920 series) was placed two centimeters distal to the antecubital fold and was then inflated to ≤250 mmHg for 5 min. Prior to the cuff release, measurements were recorded for 20 s, following release post-occlusion measurements were recorded for 3 min.

Validated software (Medical Imaging Applications, LLC., Coralville, IA, USA) was used for offline analysis of the recorded images for brachial diameter (mm) and blood velocity (shear rate). ECG gating was used for consistent cardiac cycles (end diastole) for brachial artery diameter measurements. FMD (%) was quantified as the peak diameter observed post-occlusion and reported as percent change from the average 1 min baseline diameter.

### 2.5. Statistical Analyses

Data analysis was performed using the Statistical Package for the Social Sciences (SPSS version 22.0). Normality of the data was confirmed with a Shapiro–Wilk test. Differences between obese and normal-weight groups in baseline variables were computed by independent *t* tests. A 2 (group) × 2 (treatment) × 4 (time points: Pre, Post, R1 [1 h into recovery], and R2 [two hours into recovery]) repeated measures analyses of variance (ANOVA) were utilized to examine the effect of acute aerobic exercise on serum levels of CTRP9 and FMD. Bonferroni post hoc analysis was utilized for pairwise comparisons. The. Greenhouse-Geisser correction of degrees of freedom was used when sphericity assumptions were violated. Pearson product-moment correlations were used to examine the relationship of CTRP9 with FMD. A post hoc power analysis was conducted using the program G*Power (version 3.1.9.2) for primary outcome measures. Based on the effect size ranging from 0.54 to 0.72 with an α-level of 0.05 for CTRP9 and FMD in response to both HIIE and CME, the overall sample size of 16 participants in this study achieved an adequate power (>80%). Statistical significance was defined as a *p*-value ≤ 0.05.

## 3. Results

### 3.1. Anthropometric Measurements of the Study Participants

As shown in Table 1, the analysis revealed a significant difference in age, weight, BMI, relative VO_2_ max, waist circumference, hip circumference, waist-to-hip ratio, resting systolic blood pressure, and diastolic blood pressure between obese and normal-weight participants.

### 3.2. Measurement of Serum CTRP9 and Flow-Mediated Dilation

**Figure 1:**
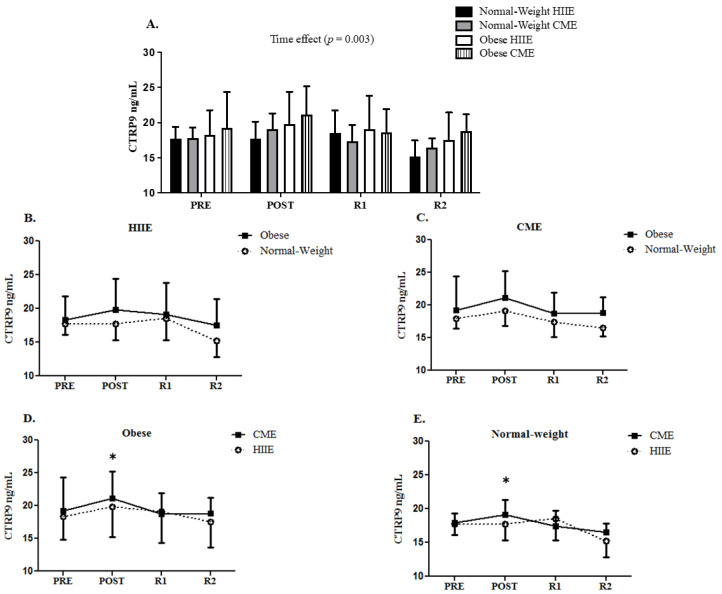
The response of serum CTRP9 following HIIE vs. CME in normal-weight and obese participants (panel (**A**)). The CTRP9 response to HIIE in normal-weight and obese participants (panel (**B**)). The CTRP9 response to CME in normal-weight and obese participants (panel (**C**)). The comparison of CTRP9 response to CME vs. HIIE in obese participants (panel (**D**)). The comparison of CTRP9 response to CME vs. HIIE in normal-weight participants (panel (**E**)). Data are presented as means ± SD. * Time effect vs. PRE.

**Figure 2:**
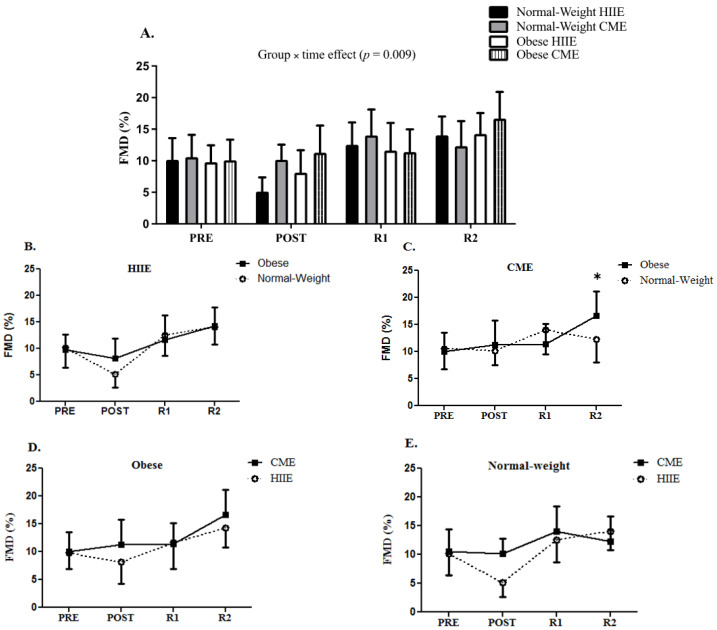
The FMD response to HIIE vs. CME in normal-weight and obese participants (panel (**A**)). The FMD response to HIIE in normal-weight and obese participants (panel (**B**)). The FMD response to CME in normal-weight and obese participants (panel (**C**)). The comparison of FMD response to CME vs. HIIE in obese participants (panel (**D**)). The comparison of FMD response to CME vs. HIIE in normal-weight participants (panel (**E**)). Data are presented as means ± SD. * Group × time effect.

At baseline, no difference was observed in CTRP9 and FMD between obese and normal-weight participants. However, the obese participants had greater brachial artery diameters than the normal-weight participants at baseline for CME (4.5 mm vs. 3.8 mm, *p* = 0.021) but not HIIE (4.3 mm vs. 3.9 mm, *p* = 0.064). Repeated measures ANOVA demonstrated a significant time effect for CTRP9 immediately following acute HIIE and CME in both groups (F _[3, 42]_ = 5.435, *p* = 0.003) (see Figure 1). Furthermore, a group by time interaction for FMD was observed (F _[3, 42]_ = 4.346, *p* = 0.009), with a greater CME-induced FMD response at two hours into recovery in obese participants than normal-weight participants (see Figure 2).

### 3.3. Correlations between CTRP9 and FMD

**Figure 3:**
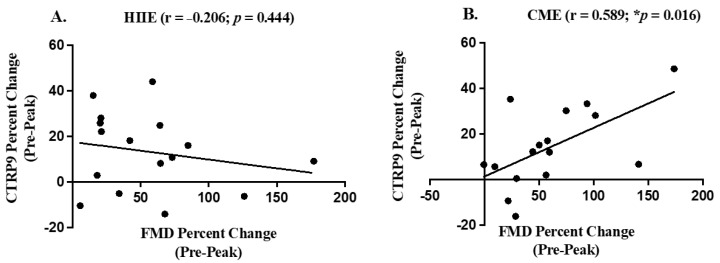
The relationship between CTRP9 percent change (pre-peak value) and FMD percent change (pre-peak value) using Pearson correlations in response to HIIE (panel (**A**)) and CME (panel (**B**)). * Indicates significant Pearson correlation.

When combined with both obese and normal-weight participants, our analyses did not observe a significant correlation between CTRP9 and FMD at baseline. Moreover, a positive Pearson correlation in percent change (baseline to peak value) between CTRP9 and FMD was found following acute CME (r = 0.589, *p* = 0.016) (see Figure 3B). However, this relationship between CTRP9 and FMD following HIIE failed to exist (r = −0.206, *p* = 0.444) (see Figure 3A). Finally, when the relationship of CTRP9 with FMD was analyzed in either obese or normal-weight group alone, our analyses did not show any significant results.

## 4. Discussion

We examined the effect of acute HIIE vs. CME on serum CTRP9 and brachial artery FMD responses in obese and normal-weight participants. Our results demonstrated that obese participants elicited a similar elevation in serum CTRP9 immediately following both acute HIIE and CME compared to normal-weight participants. Furthermore, a greater elevation in FMD was only found at 2 h into recovery following acute CME in obese participants when compared to normal-weight participants. These findings support the use of CME in this population as an effective modality to improve cardiovascular health, as evidenced by increased FMD in our participants with obesity.

While this study did not observe a difference at the baseline level of CTRP9 between obese and normal-weight participants, the literature regarding CTRP9 and obesity remains debated. Specifically, research has previously shown that the circulating level of CTRP9 was lower following a high-fat diet in obese mice when compared to lean mice [25]. While other research that utilized a CTRP9 knockout mice model demonstrated that mice became obese even when fed normal chow [26]. In humans, the level of serum CTRP9 has been shown to inversely relate to visceral fat [12]. In contrast, another study found serum levels of CTRP9 were higher in patients with obesity-associated comorbidities (e.g., diabetes, hypertension, hypercholesterolemia) than healthy normal-weight individuals, but following weight-loss surgery there was decrease in CTRP9 [14]. While lower serum CTRP9 levels in metabolically unhealthy individuals were observed [12], participants in our study did not have a history of metabolic syndrome or diseases.

To the best of our knowledge, this study is the first to examine how obesity might influence the release of circulating CTRP9 in response to acute exercise (HIIE or CME), although no difference was found between obese and normal-weight groups. In agreement with our findings, previous research demonstrated an elevation in CTRP9 following acute HIIE in normal-weight participants [22]. However, the literature has previously reported that CTRP9 plays a compensatory role in obesity and arterial stiffness [14,27]. Specifically, CTRP9 promotes vasodilation through eNOS activation via the AMPK-Akt-eNOS mechanism in human umbilical vein endothelial cells [9]. The expression of increased CTRP9 in pulmonary epithelial cells enhanced the activation of eNOS and reduced the release of vasoconstricting factor, such as endothelin-1 [28]. Furthermore, CTRP9 can attenuate cytokine-induced vascular inflammation in endothelial cells via AMPK activation, including a reduction in TNF-α induced activation of inflammatory transcription factor (NF-kB) and adhesion molecules (ICAM-1 and VCAM-1) [27]. Thus, further studies are warranted to discover the effect of exercise training (either HIIE or CME) to gain a better understanding of how CTRP9 might play a potential compensatory role in the improvement of cardiovascular health in obesity-associated metabolic complication, such as insulin resistance [12,14]. As HIIE is a time-effective strategy to reduce body fat percentage in adults with obesity [29] and has been demonstrated to be more enjoyable than CME [30].

The measurement of FMD provides prognostic information to possibly exceed the assessment of traditional risk factors for cardiovascular events [31], although there is a debate regarding the normalization for FMD stimulus (shear rate) [6]. Our laboratory has recently demonstrated that the normalization of FMD (using FMD [%]/shear rate [area under the curve]) did not change the results for the acute response to aerobic exercise using a subset of the participants from this study [32]. As such, the presentation of FMD (%) was utilized in the current study as it is clinically relevant [33]. Importantly, obesity has been associated with the impairment of FMD [34]; however, the present study did not observe any baseline difference with normal-weight individuals in agreement with the findings by Hallmark et al., 2014. This discrepancy might be due to the utilization of different age groups: older adults by Davison and colleagues vs. young adults in the current study. Interestingly, our results demonstrated a significant interaction for group by time effect in FMD, with a greater CME-induced response in obese than normal-weight participants, which remained elevated two hours into recovery. These findings may support the use of acute CME to improve endothelial function in individuals with obesity [35]. Specifically, enhanced endothelial function in response to acute CME may be a result of the exercise-mediated shear stress on the endothelium to enhance NO bioavailability [35]. While this study is the first to examine the impact of obesity on FMD response following acute HIIE vs. CME, acute HIIE has been demonstrated to improve FMD equally when compared to CME in patients with coronary artery disease [36]. However, Currie et al. 2012 reported a greater total workload in acute CME than HIIE, whereas this study equated total work (caloric expenditure) between exercise conditions (HIIE and CME) in both groups, which may potentially explain the variance in this finding. Finally, a significant positive relationship in percent change (baseline to peak value) between CTRP9 and FMD was found following acute CME, providing evidence of enhanced endothelial function in both groups. However, additional investigation is needed to further verify the relationship of exercise-mediated CTRP9 and endothelial function in older adults with obesity. Additionally, the impact of sex on these findings should be investigated as only males were included in this study. The use of CTRP9 could potentially predict the effectiveness of exercise treatments to prevent or delay obesity-associated cardiovascular events.

This study is not without limitations. For example, there was an age difference between groups, with the obese participants being slightly older than normal-weight participants. Additionally, our findings are limited to young healthy males, which may not be representative of females and or the general population. Lastly, we did not directly measure adiposity, as we classified our participants for obesity using body mass index and waist-to-hip ratio. Future research should utilize a larger sample size with both males and females and directly measure adiposity, to confirm our findings regarding endothelial function and CTRP9 in response to HIIE and CME in participants with obesity.

## 5. Conclusions

In conclusion, this study demonstrated that participants with obesity exhibited a similar CTRP9 response following both acute HIIE and CME compared to participants of normal-weight. Furthermore, a greater FMD elevation was observed in obese participants in response to CME when compared to normal-weight participants. While HIIE is a time-effective strategy to improve metabolic health and inflammation [37,38], the novel results from this study provide a foundation for additional examination of the mechanisms of exercise-mediated CTRP9 on endothelial function in individuals with obesity.

## Figures and Tables

**Table 1:** Characteristics of Participants.

Variable	Normal-Weight n = 7	Obese n = 9	*p*-Value
Age (years)	23 ± 2	28 ± 5	0.019
Height (m)	1.79 ± 0.04	1.78 ± 0.06	0.815
Weight (kg)	72 ± 10	116 ± 18	<0.001
Body Mass Index (kg/m^2^)	22 ± 2	36 ± 4	<0.001
Waist (cm)	80 ± 7	113 ± 16	<0.001
Hip (cm)	96 ± 5	118 ± 8	<0.001
WHR (a.u.)	0.84 ± 0.06	0.95 ± 0.09	0.009
VO_2_ max (mL/kg/min)	51 ± 5	37 ± 6	<0.001
Resting Heart Rate (bpm)	67 ± 10	73 ± 5	0.148
rSBP (mmHg)	116 ± 7	138 ± 12	<0.001
rDBP (mmHg)	72 ± 5	85 ± 8	<0.001

Data are represented as mean ± SD. WHR = waist-to-hip ratio; VO_2_ max = maximal oxygen consumption; rSBP = resting systolic blood pressure; rDBP = resting diastolic blood pressure.

## Data Availability

Not applicable.

## Abbreviations

AMPK	AMP-activated protein kinase
eNOS	Endothelial cell nitric oxide synthase
ANOVA	Analyses of variance
FMD	Flow-mediated dilation
BMI	Body mass index
HIIE	High-intensity interval exercise
CME	Continuous moderate-intensity exercise
NO	Nitric oxide
CTRP9	C1q-TNF-related protein-9
RPE	Rating of perceived exertion
EDTA	Ethylenediaminetetraacetic acid
SST	serum separation tube
ELISA	Enzyme-linked immunosorbent assay
VO_2_ max	Maximal oxygen consumption
